# Discrimination of Wild *Paris* Based on Near Infrared Spectroscopy and High Performance Liquid Chromatography Combined with Multivariate Analysis

**DOI:** 10.1371/journal.pone.0089100

**Published:** 2014-02-18

**Authors:** Yanli Zhao, Ji Zhang, Tianjun Yuan, Tao Shen, Wei Li, Shihua Yang, Ying Hou, Yuanzhong Wang, Hang Jin

**Affiliations:** 1 Institute of Medicinal Plants, Yunnan Academy of Agricultural Sciences, Kunming, Yunnan, the People's Republic of China; 2 Yunnan Reascend Tobacco Technology (Group) Co., Ltd., Kunming, Yunnan, the People's Republic of China; 3 College of Resources and Environment, Yuxi Normal University, Yuxi, Yunnan, the People's Republic of China; National Research Council of Italy, Italy

## Abstract

Different geographical origins and species of *Paris* obtained from southwestern China were discriminated by near infrared (NIR) spectroscopy and high performance liquid chromatography (HPLC) combined with multivariate analysis. The NIR parameter settings were scanning (64 times), resolution (4 cm^−1^), scanning range (10000 cm^−1^∼4000 cm^−1^) and parallel collection (3 times). NIR spectrum was optimized by TQ 8.6 software, and the ranges 7455∼6852 cm^−1^ and 5973∼4007 cm^−1^ were selected according to the spectrum standard deviation. The contents of polyphyllin I, polyphyllin II, polyphyllin VI, and polyphyllin VII and total steroid saponins were detected by HPLC. The contents of chemical components data matrix and spectrum data matrix were integrated and analyzed by partial least squares discriminant analysis (PLS-DA). From the PLS-DA model of NIR spectrum, *Paris* samples were separated into three groups according to the different geographical origins. The R^2^X and Q^2^Y described accumulative contribution rates were 99.50% and 94.03% of the total variance, respectively. The PLS-DA model according to 12 species of *Paris* described 99.62% of the variation in X and predicted 95.23% in Y. The results of the contents of chemical components described differences among collections quantitatively. A multivariate statistical model of PLS-DA showed geographical origins of *Paris* had a much greater influence on *Paris* compared with species. NIR and HPLC combined with multivariate analysis could discriminate different geographical origins and different species. The quality of *Paris* showed regional dependence.

## Introduction

Traditional Chinese medicine (TCM) is gaining greater acceptance throughout the world, especially in western countries, for improving health and preventing or healing diseases [1]. It is well known that TCM are composed of animal drugs, medicinal plants, fungi and minerals, among which medicinal plants play an important role for their wealthy sources, rich species and diverse components [2]. These plants have been used to treat various diseases for thousand years in Eastern Asia [3]. Recently, a large number of bioactive components and metabolites were found from medicinal plants, which are considered as the key ingredients of TCM development and utilization [4]. However, the quality and contents of bioactive components in medicinal plants are extremely variable depending on species, geographical origins, cultivation, growth altitude, soil, harvest time and climate conditions such as temperature, sun exposure time and rainfall [5], [6]. The clarification of the source and species of medicinal plants plays the decisive role in the quality control of TCM formulas, which is the fundamental prerequisite for its worldwide recognition and acceptance.

**Figure 1:**
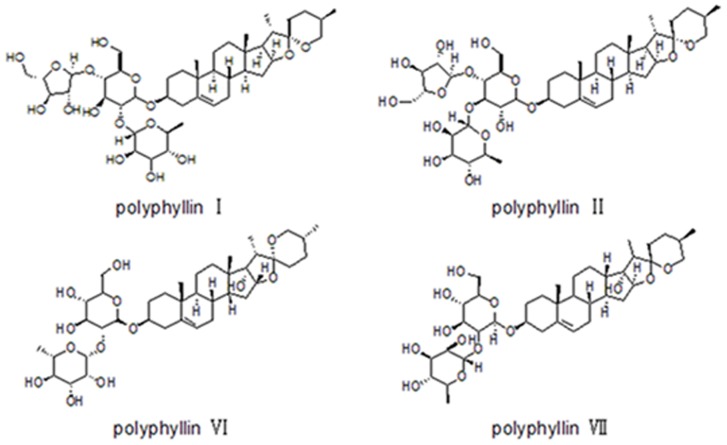
Chemical structures of polyphyllin I, II, VI and VII.

*Paris*, belonging to the family Liliaceae, contains about 24 species and mainly distributes in Europe and Eastern Asia. There are 22 species of *Paris* in China, and the diversity center of *Paris* is located in Southwest China [7]. The dried rhizome paridis is the main raw material of Chinese patent drugs “Yunnan Baiyao”, “GongXue Ning”, and “Jidesheng snake tablet” [8]. The phytochemistry research indicates that abundant active ingredients including steroidal saponins, flavonoids, fatty acid ester and endophytic fungi are in the dried rhizome of *Paris*, and steroid saponins such as polyphyllin I, polyphyllin II, polyphyllin VI, and polyphyllin VII are the most investigated ones [8], [9], [10], [11]. Their chemical structures are depicted in Figure 1. Modern pharmacology has demonstrated that polyphyllin has powerful pharmacological activities on stypticity, spermicide, homeostasis, analgesic, and as a potential anti-cancer drug for the functions of cytotoxicity and induction of apoptosis [12], [13], [14].

In folk of China, several species of *Paris* plants have a history used to treat snakebite, hemostasis, fractures, parotitis and abscess [8]. However, only the rhizomes of *P. polyphylla* var. *chinensis* and *P. polyphylla* var. *yunnanensis* are officially recorded in Chinese Pharmacopoeia. As we know, the morphological characteristics are similar between close related species. It is much more difficult to discriminate dry rhizomes of the same genus by traditional morphological identification method, especially the original powder. On the other hand, common methods like microscopic identification, thin layer chromatography are laborious and time-consuming.

In recent years, near infrared (NIR) spectroscopic methods have been used in analysis of vegetable, fruit, coffee, green tea, wine, plant and pharmaceutical [15], [16], [17], [18], [19], [20], [21]. High performance liquid chromatography (HPLC) is considered as a robust method in numerous applications of qualitative and quantitative analyses of TCM for its easy operation, high accuracy and wide suitability. In quantification, determination of a group of principal active constituents with similar or different structures in one medicinal plant has been widely implemented [22], [23], [24], [25].

In this research, NIR and HPLC in combination with multivariate statistical analysis were applied for discriminating *Paris* plants of different species and different origins quantitatively and qualitatively, and also found the key factor of identification of *Paris*.

## Materials and Methods

### Materials

**Figure 2:**
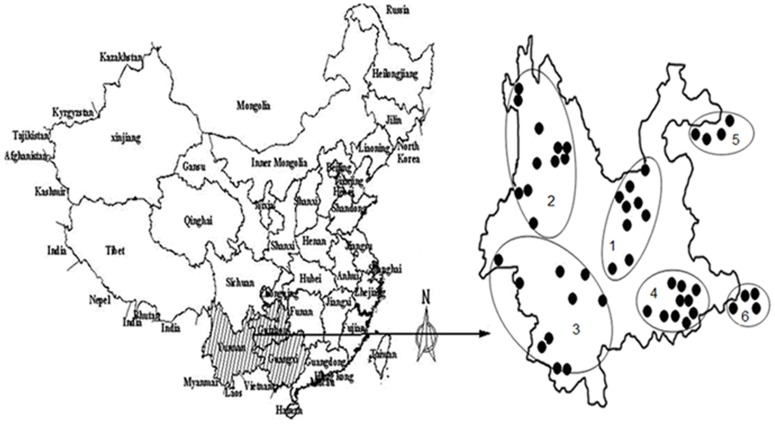
The wild *Paris* collected from main geographical origins in China. **1**: Central Yunnan (16#, 18#, 19#, 21#, 26#, 32#, 33#, 37#, 43#); **2**: Northwestern Yunnan (9#, 10#, 11#, 12#, 17#, 24#, 28#, 36#, 38#, 39#, 46#); **3**: Southwestern Yunnan (22#, 25#, 27#, 30#, 35#, 40#, 41#, 44#, 47#, 48#); **4**: Southeastern Yunnan (1#, 3#, 4#, 13#, 20#, 23#, 29#, 31#, 34#, 42#); **5**: Guizhou (2#, 6#, 7#, 8#); **6**: Guangxi (5#, 14#, 15#, 45#).

Forty eight samples of wild *Paris* including 12 species were collected from three main distribution areas in southwest China: Yunnan, Guizhou and Guangxi Provinces (Figure 2). They were identified and authenticated by Doctor J.Y. Zhang, Yunnan Academy of Agricultural Sciences (Table 1). The herbariums were preserved in the institute of medicinal plants, Yunnan academy of agricultural sciences. The rhizomes of *Paris* plants were dried at the temperature of 50°C, and then ground to fine powder and storaged in the zip lock bags until further analysis. No specific permits were required for the described field studies, as no endangered or protected species were sampled, and the localities where the samples came from are not protected in any way.

**Table 1:** Sample species and locations.

Sample number		Species	Location	Sample number	Species	Location	
1#		*P. caobangensis*	Wenshan, Yunnan	25#	*P. polyphylla* var. *yunnanensis*	Puer, Yunnan	
2#		*P. cronquistii*	Bijie, Guizhou	26#	*P. polyphylla* var. *yunnanensis*	Yuxi, Yunnan	
3#		*P. cronquistii*	Wenshan, Yunnan	27#	*P. polyphylla* var. *yunnanensis*	Lincang, Yunnan	
4#		*P. cronquistii* var. *xichouensis*	Wenshan, Yunnan	28#	*P. polyphylla* var. *yunnanensis*	Nujiang, Yunnan	
5#		*P. delavayi* var. *petiolata*	Baise, Guangxi	29#	*P. polyphylla* var. *yunnanensis*	Wenshan, Yunnan	
6#		*P. fargesii*	Bijie, Guizhou	30#	*P. polyphylla* var. *yunnanensis*	Puer, Yunnan	
7#		*P. fargesii*	Bijie, Guizhou	31#	*P. polyphylla* var. *yunnanensis*	Gejiu, Yunnan	
8#		*P. fargesii*	Bijie, Guizhou	32#	*P. polyphylla* var. *yunnanensis*	Yuxi, Yunnan	
9#		*P. mairei*	Dali, Yunnan	33#	*P. polyphylla* var. *yunnanensis*	Kunming, Yunnan	
10#		*P. polyphylla*	Dali, Yunnan	34#	*P. polyphylla* var. *yunnanensis*	Wenshan, Yunnan	
11#		*P. polyphylla*	Lijiang, Yunnan	35#	*P. polyphylla* var. *yunnanensis*	Puer, Yunnan	
12#		*P. polyphylla var. alba*	Dali, Yunnan	36#	*P. polyphylla* var. *yunnanensis*	Nujiang, Yunnan	
13#		*P. polyphylla* var. *chinensis*	Wenshan, Yunnan	37#	*P. polyphylla* var. *yunnanensis*	Kunming, Yunnan	
14#		*P. polyphylla* var. *chinensis*	Baise, Guangxi	38#	*P. polyphylla* var. *yunnanensis*	Nujiang, Yunnan	
15#		*P. polyphylla* var. *chinensis*	Baise, Guangxi	39#	*P. polyphylla* var. *yunnanensis*	Nujiang, Yunnan	
16#		*P. polyphylla* var. *pseudothib*	Dongchuan, Yunnan	40#	*P. polyphylla* var. *yunnanensis*	Xishuangbanna, Yunnan	
17#		*P. polyphylla* var. *yunnanensis*	Baoshan, Yunnan	41#	*P. polyphylla* var. *yunnanensis*	Lincang, Yunnan	
18#		*P. polyphylla* var. *yunnanensis*	Kunming, Yunnan	42#	*P. polyphylla* var. *yunnanensis*	Wenshan, Yunnan	
19#	*P. polyphylla* var. *yunnanensis*		Kunming, Yunnan	43#	*P. polyphylla* var. *yunnanensis*	Kunming, Yunnan	
20#	*P. polyphylla* var. *yunnanensis*		Wenshan, Yunnan	44#	*P. vietnamensis*	Lincang, Yunnan	
21#	*P. polyphylla* var. *yunnanensis*		Chuxiong, Yunnan	45#	*P. cronquistii* var. *xichouensis*	Baise, Guangxi	
22#	*P. polyphylla* var. *yunnanensis*		Puer, Yunnan	46#	*P. mairei*	Nujiang, Yunnan	
23#	*P. polyphylla* var. *yunnanensis*		Wenshan, Yunnan	47#	*P. polyphylla* var. *yunnanensis*	Xishuangbanna, Yunnan	
24#	*P. polyphylla* var. *yunnanensis*		Nujiang, Yunnan	48#	*P. polyphylla* var. *yunnanensis*	Dehong, Yunnan	

### Instruments and Reagents

The standards (polyphyllin I, polyphyllin II, polyphyllin VI, and polyphyllin VII) were purchased from the National Institute for Control of Pharmaceutical and Biological Products (Beijing, China). The purity of all the standards was greater than or equal to 98%. HPLC grade acetonitrile and methanol were obtained from TEDIA (Ohio, USA). Purified water (HPLC grade) was produced by Milli-Q system (Massachusetts, USA). Other reagents were all of analytical grade.

HPLC system (Shimadzu Technologies, Kyoto, Japan) was equipped with Workstation software class-VP (Shimadzu Technologies) for recording chromatograms and composed of HPLC-10 integrator, HPLC-10ATVP pump, and SDP-M10A VP detector (DAD). All chromatographic separations were carried out on a Shim-pack VP-ODS C18 (150×4.6 mm, particle size: 5 µm) from Shimadzu (Kyoto, Japan). Antaris II Fourier Transform Near Infrared Spectroscopy (Thermo Fisher Scientific INC., USA) was attached with diffuse reflection module. The spectrum collecting software Result™ 2.1 and the analysis software TQ 8.6 included in the instrument were employed. Traditional Chinese medicine grinder DFT-100 (Zhejiang wenling Linda machinery co., LTD) was applied. Stainless steel sieve tray 80 mesh (Tai'an of Chinese and western, Beijing) was used. The multivariate data analysis software was SIMCA-P 11.0 (Umetrics, Umea, Sweden).

### Sample Preparation for NIR Analysis

The rhizomes powder (20.0 g) was weighed before it was sufficient mixed, then transferred to the sample cup of NIR and compressed. Collecting the spectrum of NIR by diffuse reflection module of Result™ 2.1. The parameter settings were scanning (64 times), resolution (4 cm^−1^), scanning range (10000 cm^−1^∼4000 cm^−1^) and parallel collection (3 times).

### Sample Preparation for HPLC Analysis

The *Paris* rhizomes powder (0.5000 g) was extracted with 25 mL alcohol under refluxing for 45 min. After cooling to room temperature, 1.5 mL of the extract was transferred into 2 mL centrifuge tube, centrifuged at 16,000 rpm for 10 min, and then reserved supernatant for detection. The contents of polyphyllin I, polyphyllin II, polyphyllin VI, and polyphyllin VII and total steroid saponins were detected by HPLC. The mobile phase solvents, flow-rate, injection volume, column temperature and detection wavelength have been optimization by the reference of Zhang, et al [11].

### Data Preprocessing

The NIR spectrums of *Paris* were preprocessed with Norris, mean centering, standardization, and second derivative successively by software TQ 8.6. The stability of 25 times parallel collections of a sample (30 #) was considered in the 95% confidence by SIMCA-P software 11.0.

The NIR resulting .spc files were converted to .csv data files by the multivariate statistical analysis of SIMCA-P software 11.0. The HPLC resulting .xls files were converted to .csv data files by the Excel software. Then the .csv files were imported to multivariate statistical analysis of SIMCA-P software 11.0. Different geographical origins and species of wild *Paris* were identified by partial least squares discriminant analysis (PLS-DA) according to the NIR spectra and the contents of chemical compositions. PLS was used to visualize general clustering, trends, and outliers among the observations.

### Multivariate Analysis of NIR and HPLC

NIR spectrum was optimized by TQ 8.6 software, and the ranges 7455∼6852 cm^−1^ and 5973∼4007 cm^−1^ were selected according to the spectrum standard deviation. The higher the spectra standard deviation was, the greater the contribution to classification. The contents of chemical components data matrix and spectrum data matrix were integrated and analyzed by PLS-DA. PLS-DA was applied to obtain the first understanding of relationships between the data matrix, and to examine the differences in the spectrum of different geographical origins and species of *Paris*. The efficiency and reliability of the PLS-DA model were verified by percent variation of the x and y variables explained by the model (R^2^X, R^2^Y) and the predictive performance of the model (Q^2^) [26].

## Results and Discussion

### Validation of the NIR Spectroscopy and HPLC methods

**Figure 3:**
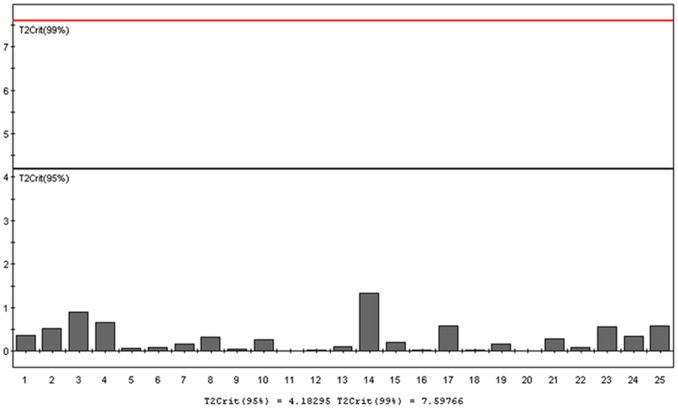
Stability control chart of NIRS of *Paris*.

The precision of the NIR spectrometer was tested by assaying the same sample thirteen times continuously. The relative standard deviation (RSD) (*n* = 13) of precision for *Paris* powder was 0.184%. The repeatability of the NIR spectrometer was evaluated by assaying 13 replicate of the same sample. The RSD (*n* = 13) of repeatability for *Paris* powder was 0.232%. Within 3 h, the stability of sample was analyzed every 20 min. The RSD for stability was 0.297%. The spectral reproducibility is an essential factor in assessing the quality of the measurement technique. To gain insight into the reproducibility of system, 25 times parallel collections of sample 30 # were executed and evaluated by Hotelling T^2^ (Figure 3). The results showed that the parallel spectrum acquisitions possessed satisfactory stability with coefficient 4.18 and 7.58 in the 95% and 99% levels, respectively. The results indicated that NIR was a reliable method for discriminant analysis.

The precision of HPLC was evaluated by analyzing the same sample extract six times continuously. The RSD (*n* = 6) of precision were 1.260%, 0.752% and 2.182% for polyphyllin I, polyphyllin II and polyphyllin VII, respectively. The repeatability of HPLC was tested by assaying 6 replicate of the same sample extract. The RSD (*n* = 6) of repeatability for *Paris* extract were 1.384%, 0.941% and 2.534% for polyphyllin I, polyphyllin II and polyphyllin VII, respectively. Stability of sample extract was tested every 4 h within 48 h, and the RSD were 1.346%, 1.225% and 2.734% for polyphyllin I, polyphyllin II and polyphyllin VII, respectively. The results indicated good performance of the method for HPLC assay.

### Characterization of the Spectrum and Contents of Chemical Compositions

**Figure 4:**
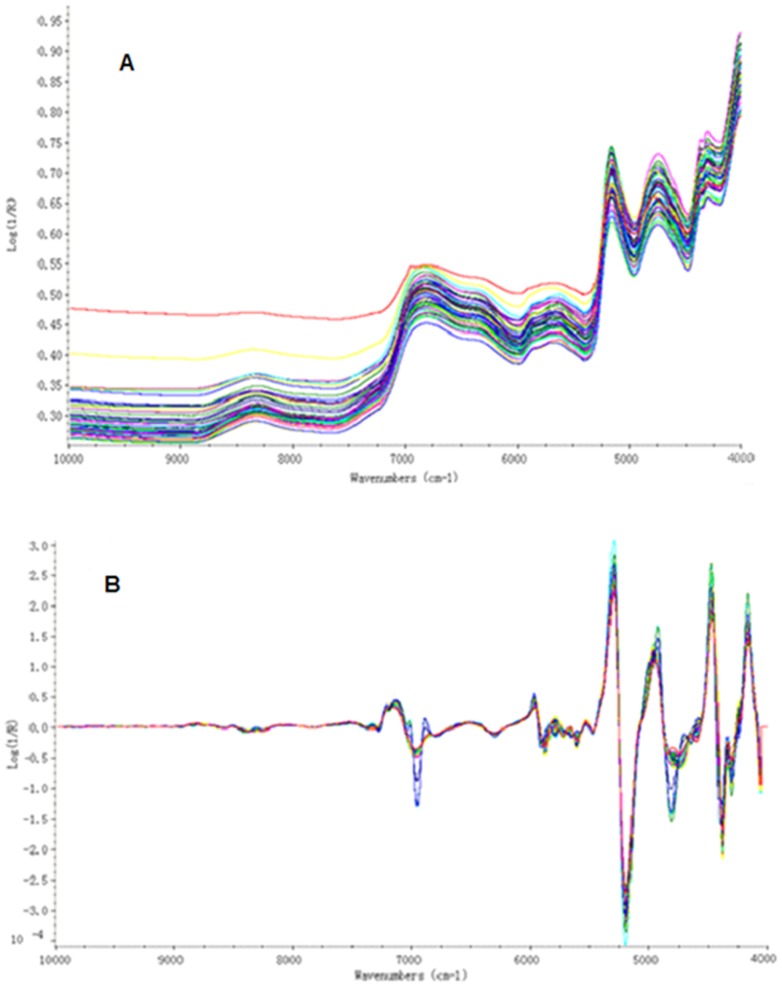
The original (A) and second derivative (B) NIR spectra of *Paris*.

NIR spectrum and the chemical components contents of *Paris* were shown in Figure 4 and Table 2, respectively. Figure 4A showed the original spectra collected for the *Paris* samples, which illustrated the lowest molecular absorptivities were in the region 10000–7515 cm^−1^, with higher values in the region 7150–5436 cm^−1^ and still higher absorbance levels in the region 5326–4045 cm^−1^. The wavelength at 8380–8230 cm^−1^ corresponds to C–H second overtone stretch vibration modes in CH_3_ and CH_2_ groups, whereas the bands located between 6900 and 6800 cm^−1^ are the first overtone of O–H bands. In Figure 4B, the wavelengths at 7145 and 6953 cm^−1^ are related to C–H combination bands in CH_2_. The absorption band at 5181 cm^−1^ is assigned to polysaccharide combination band of O–H stretch vibration and the transformation of HOH. The wavelength at 4400 cm^−1^ is the combination band of O–H and C–O stretch vibration in glucose. In HPLC analysis, the retention time of polyphyllin I, polyphyllin II, polyphyllin VI and polyphyllin VII were at 34.828 min, 32.384 min, 23.048 min and 20.980 min, respectively. According to the results of HPLC, we found that polyphyllin VI only was detected in sample 17#. Based on this point, the compound polyphyllin VI was not used for discrimination analysis. Chemical components including polyphyllin I, polyphyllin II, polyphyllin VII, and total steroid saponins were employed in discrimination analysis, with “0” expressed the one could not be determined.

**Table 2:** Contents of detected components in *Paris* (dry weight, mg·g^−1^).

Sample number	polyphyllin I	polyphyllin II	polyphyllin VI	polyphyllin VII	Total steroid saponins
1	8.61	-	-	0.49	9.11
2	11.53	-	-	0.14	11.67
3	-	-	-	0.15	0.15
4	21.56	5.06	-	0.56	27.18
5	3.32	-	-	1.63	4.95
6	4.45	1.23	-	-	5.67
7	12.57	0.90	-	0.15	13.63
8	-	2.66	-	0.43	3.09
9	10.03	1.34	-	0.43	11.81
10	15.76	2.55	-	0.16	18.47
11	8.41	0.91	-	1.28	10.60
12	9.67	1.69	-	0.83	12.19
13	27.04	-	-	4.32	31.36
14	11.00	1.67	-	0.25	12.91
15	12.09	1.24	-	0.33	13.66
16	31.03	1.91	-	0.40	33.34
17	3.34	-	2.97	0.15	6.55
18	2.90	-	-	0.43	3.33
19	9.24	1.49	-	-	10.73
20	5.00	1.65	-	0.57	7.22
21	6.92	-	-	0.68	7.60
22	12.41	1.59	-	0.15	14.15
23	13.04	4.69	-	0.69	18.80
24	16.37	2.27	-	0.17	18.80
25	2.91	-	-	0.25	3.16
26	13.44	3.41	-	0.51	17.36
27	19.80	2.04	-	0.24	22.07
28	-	-	-	0.16	0.16
29	-	3.75	-	0.42	4.18
30	18.35	1.90	-	0.42	20.66
31	14.52	1.47	-	0.25	16.24
32	3.81	-	-	0.62	4.43
33	3.43	-	-	1.54	4.97
34	-	2.59	-	0.30	2.89
35	11.63	3.24	-	0.87	15.74
36	4.99	1.74	-	0.17	6.90
37	11.04	2.93	-	0.74	14.71
38	9.85	-	-	0.43	10.28
39	4.39	-	-	0.54	4.93
40	15.60	4.37	-	0.25	20.22
41	10.57	3.85	-	0.15	14.56
42	15.02	-	-	0.21	15.23
43	12.93	1.30	-	0.52	14.75
44	3.63	-	-	0.54	4.18
45	9.15	5.53	-	0.40	15.08
46	20.16	3.16	-	0.15	23.46
47	-	-	-	0.25	0.25
48	16.57	1.75	-	0.32	18.64

Note: “-” means undetected.

### Discriminant Analysis of *Paris* from Different Geographical Origins by NIR Spectrum and HPLC

**Figure 5:**
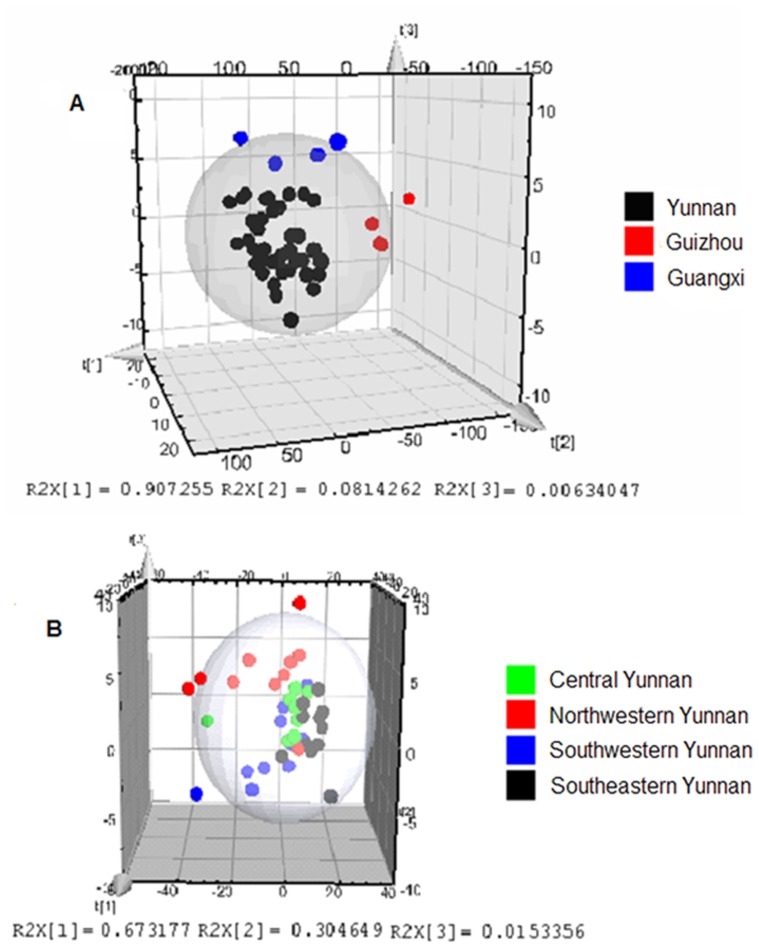
PLS model of *Paris* from Yunnan, Guizhou and Guangxi Provinces (A) and different geographical origins from Yunnan Provinces (B).

According to the diversity of NIR spectrum, stepwise discriminant analysis of PLS was utilized to analyze the samples from different geographical origins. In Figure 5A, forty eight collections of *Paris* samples were obviously separated into three groups according to the different geographical origins. The samples from Yunnan Province were clearly separated from the other two regions. The R^2^X and Q^2^Y described accumulative contribution rates were 99.50% and 94.03% of the total variance, respectively.

In further insight into samples from Yunnan Province for their multiple distribution areas, forty samples were separated into four groups. In Figure 5B, the R^2^X and Q^2^Y described accumulative contribution rates were 99.28% and 94.32% of the total variance, respectively. However, three samples 3#, 24# and 37# were classified incorrectly. The reason for that could not be found.

**Figure 6:**
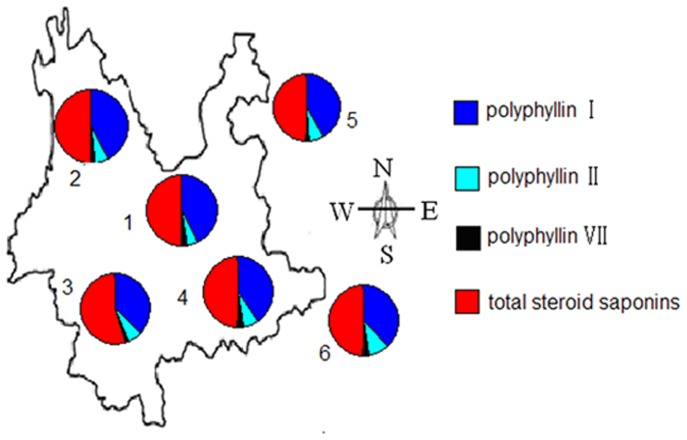
The average contents of polyphyllin I, II, VII and total steroid saponins in *Paris* from different geographical regions: (1) Central Yunnan; (2) Northwestern Yunnan; (3) Southwestern Yunnan; (4) Southeastern Yunnan; (5) Guizhou; (6) Guangxi.

Furthermore, the average contents of chemical components (polyphyllin I, polyphyllin II, polyphyllin VII, and total steroid saponins) were used for the contribution of the geographical origins (Figure 6). More interestingly, the variation of chemical components coupling with the locations of wild *Paris* samples from Yunnan (Central, Northwestern, Southwestern and Southeastern), Guizhou and Guangxi Provinces in the pie charts showed the visualization of the major differences among the six geographical origins of samples. The samples from Southwestern Yunnan had the highest contents of polyphyllin I (11.147 mg·g^−1^) and total steroid saponins (13.363 mg·g^−1^), while samples collected from Guangxi Province and Southeastern Yunnan had the highest contents of polyphyllin II (2.110 mg·g^−1^) and polyphyllin VII (0.796 mg·g^−1^), respectively. The contents of polyphyllin I, polyphyllin II, polyphyllin VII, and total steroid saponins in samples from Guizhou Province were all the lowest. However, there was no significant difference among the contents of polyphyllin I, polyphyllin II, polyphyllin VII, and total steroid saponins in samples from Yunnan (Central, Northwestern, Southwestern and Southeastern), Guizhou and Guangxi Provinces (*p*>0.05).

Based on the above analysis, samples from different geographical origins were different performance both in the NIR spectra and the chemical components, which might be effected by the main factors including geographical conditions, temperature, and rainfall capacity in different areas. Yunnan Province locates in southwest China and is influenced by a low latitude plateau, mountainous country monsoon climate [26]. Otherwise, Yunnan Province belongs to obvious characteristics of mountain climate with noticeable vertical climatic belt. Central and southeastern Yunnan are mainly the middle and north subtropical area, while northwest Yunnan belongs to the temperate zone, and southwestern Yunnan belongs to south subtropical [27]. In the recent years, the temperature of central, northwestern and southwestern Yunnan has increased remarkably, but the rainfall amount has decreased evidently [28]. Guizhou Province locates in northeast of Yunnan Province and is influenced by a subtropical plateau monsoon climate, the average temperature is not as stable as that of Yunnan. Guangxi Province locates in southeast of Yunnan Province and is influenced by a subtropical monsoon climate [26]. Su and Zhang [29] analyzed the relation between the photosynthesis of *Paris polyphylla* var. *yunnanensis* and the environmental factors and found that the leaf temperature increased from 11°C to 20°C, the net photosynthetic rate increased; but with the increase of temperature from 20°C to 35°C, the rate decreased. The optimal temperature was from 16°C to 28°C. With the increase of relative humidity from 20%–85%, the net photosynthetic rate increased. The optimal humidity was over 75% [29]. The results indicated the quality of *Paris* showed geographic and habitat dependencies to some extent.

### Discriminant Analysis of Different Species by NIR Spectrum and HPLC

**Figure 7:**
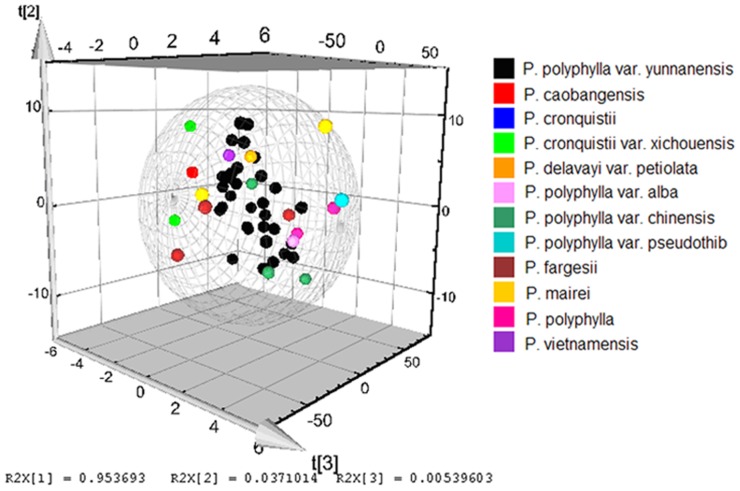
PLS model of different species of *Paris*.

Utilized PLS-DA analysis to give us a preliminary overview of similarities and differences among the species, the results suggested that wild *Paris* species impose a significant effect on the NIR spectrum. In the established PLS-DA model (Figure 7), three significant spectra data described 99.62% of the variation in X (R^2^X = 0.9962) and predicted 95.23% in Y (Q^2^Y = 0.9523) according to cross-validation. Forty-eight collections including 12 species were partly separated into different groups, *P. cronquisistii* var. *xichouensis*, *P. caobangensis*, *P. cronquistii*, *P. polyphylla* var. *alba* and *P. polyphylla* var. *pseudothib* were obviously separated from the other species. Different species of *Paris* presented certain different information in the NIR spectra, which might be according to the different chemical components or chemical constituents in the samples for their different absorption band in NIR spectrum. Furthermore, we could find that different species of *Paris* contained different levels of chemical components from the Table 2. The results of analysis of variance showed the contents of total steroid saponins in different species of *Paris* were significantly different (*p*<0.05), while the levels of polyphyllin I, polyphyllin II, polyphyllin VII had no significant difference (*p*>0.05) among different species.

### Effects of Different Geographical Origins and Species for Classification

Based on above analysis, we knew that different geographical origins and species of wild *Paris* could be separated by PLS-DA model based on their NIR spectra and chemical components. Nevertheless, in order to understand either origin or species is the key factor to identify wild *Paris*, we have enthusiastically explored the same species from different geographical origins and different species from the same geographical origin.

**Figure 8:**
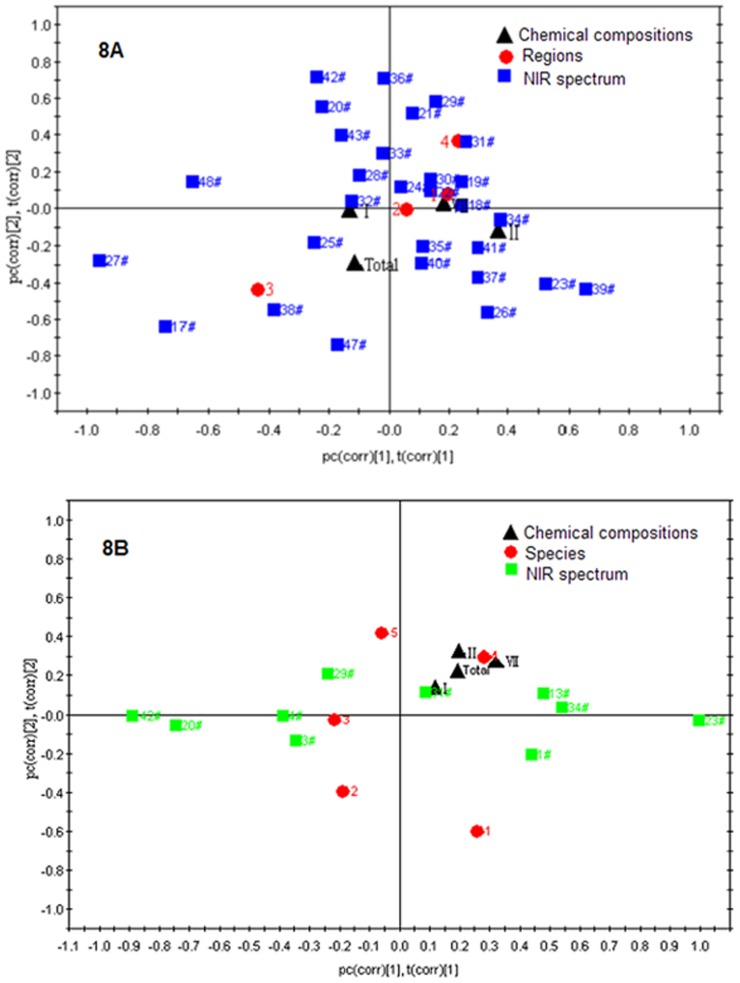
Loading Bi-plot of PLS-DA model. (A) *P. polyphylla* var. *yunnanensis* from different geographical origins. (1) Central Yunnan; (2) Northwestern Yunnan; (3) Southwestern Yunnan; (4) Southeastern Yunnan. (B) Different species of *Paris* from Southeastern Yunnan. (1) *P. caobangensis*; (2) *P. cronquistii*; (3) *P. cronquistii* var. *xichouensis*; (4) *P. polyphylla* var. *chinensis*; (5) *P. polyphylla* var. *yunnanensis*.

We selected 29 samples of *P. polyphylla* var. *yunnanensis* from four different geographical areas (central, northwestern, southwestern and southeastern) in Yunnan Province, and analyzed the effects of the geographical origins in discriminating *Pairs* by NIR spectra and chemical compositions. Loading Bi-plot of pc (corr) [1], t (corr) [1] and pc (corr) [2], t (corr) [2] generated from the loadings scaled as correlation and the scores scaled inside the correlation model of different geographical origins (Figure 8A) or different species (Figure 8B) of wild *Paris*.

From Figure 8A, pc (corr) [1], t (corr) [1] played a significant role in discriminating samples of southwestern Yunnan from the others, while pc (corr) [2], t (corr) [2] had a comparatively weak impact on separating samples of northwestern Yunnan from central and southeastern Yunnan. More interesting, the locations of samples from southwestern and southeastern Yunnan were entirely opposite, while samples from central and northwestern Yunnan were located between them. The observations are in accordance with the climate of these areas as previously stated. Furthermore, the contribution of NIR spectrum and chemical compositions of samples was given a loading value. The NIR spectrum of samples 17#, 25#, 27#, 38#, 47# and chemical components polyphyllin I and total steroid saponins, had a negative contribution to pc (corr) [1], t (corr) [1], which separated samples of southwestern Yunnan from the others, with southwestern Yunnan having a negative loading value. The results could also be found in Table 3. Although samples 17# and 38# belong to northwestern Yunnan according to administrative division, they were close to southwestern Yunnan in geographically. The above showed samples 17#, 25#, 27#, 38#, 47#, chemical components polyphyllin I and total steroid saponins were the discriminating roles for *P. polyphylla* var. *yunnanensis* from southwestern Yunnan. It is consistent with the contents determined by HPLC that the levels of polyphyllin I and total steroid saponins were highest in southwestern Yunnan, 11.15 mg·g^−1^ and 13.36 mg·g^−1^, respectively.

**Table 3:** The total score of *P. polyphylla* var. *yunnanensis* from four different geographical areas (central, northwestern, southwestern and southeastern) in Yunnan Province.

ID	t(corr)[1]	t(corr)[2]	Total score	ID	t(corr)[1]	t(corr)[2]	Total score
47#	−0.171	−0.744	19.358	Total steroid saponins	−0.115	−0.287	7.499
42#	−0.241	0.713	18.344	41#	0.299	−0.209	5.254
36#	−0.014	0.708	18.323	35#	0.114	−0.201	5.150
17#	−0.740	−0.644	17.080	25#	−0.249	−0.186	4.967
29#	0.157	0.579	15.072	28#	−0.096	0.176	4.507
26#	0.334	−0.567	14.509	30#	0.138	0.155	4.103
38#	−0.379	−0.547	14.377	19#	0.242	0.140	3.760
20#	−0.221	0.553	14.194	48#	−0.653	0.138	3.208
21#	0.078	0.515	13.378	24#	0.042	0.115	2.995
39#	0.658	−0.439	10.997	polyphyllin II	0.367	−0.112	2.701
23#	0.528	−0.410	10.323	22#	0.142	0.082	2.194
43#	−0.159	0.393	10.089	34#	0.378	−0.060	1.354
37#	0.298	−0.376	9.580	polyphyllin VII	0.184	0.029	0.849
31#	0.262	0.358	9.411	32#	−0.124	0.033	0.789
27#	−0.958	−0.284	7.882	18#	0.247	0.016	0.544
40#	0.108	−0.299	7.674	polyphyllin I	−0.130	−0.009	0.295
33#	−0.019	0.294	7.594				

Note: Total score = ABS ((t(corr)[1]*55.0%)+(t(corr)[2]*25.9%)), the first principal component explained 55.0% of total information, and the second principal component explained 25.9% of total information.

Ten samples including 5 different species of *Paris* from southeastern Yunnan were selected to understand the difference among different species from the same geographical origins by PLS-DA model. In Figure 8B, pc (corr) [1], t (corr) [1] significantly discriminated *P. caobangensis* and *P. polyphylla* var. *chinensis* from *P. cronquistii*, *P. cronquistii* var. *xichouensis* and *P. polyphylla* var. *yunnanensis*, while pc (corr) [2], t (corr) [2] separated *P. polyphylla* var. *chinensis* and *P. polyphylla* var. *yunnanensis* from *P. caobangensis*, *P. cronquistii*, and *P. cronquistii* var. *xichouensis*. Five species were located in four different quadrants, while *P. cronquistii* var. *xichouensis* was close to *P. cronquistii*, which might be the reason that *P. cronquistii* var. *xichouensis* is the variety of *P. cronquistii*, the two species have much closer genetic relationship. The NIR spectrum of samples 13#, 31#, 34# and chemical components of polyphyllin I, polyphyllin II, polyphyllin VII and total steroid saponins had a positive contribution to pc (corr) [1], t (corr) [1] and pc (corr) [2], t (corr) [2], which separated *P. polyphylla* var. *chinensis* from the others. We could clearly understand the results from Table 4. From the NIR spectrum, we could find five species of *Paris* locate in the right corresponding quadrant except *P. polyphylla* var. *yunnanensis*. In the loading Bi-plot, the closer to the origin, the smaller contribution a chemical component makes to the discrimination. The contents of polyphyllin I and total steroid saponins in *P. polyphylla* var. *chinensis* were significantly higher than in other species, they could be considered as the discriminating components for *P. polyphylla* var. *chinensis* from the other species.

**Table 4:** The total score of 5 different species of *Paris* from southeastern Yunnan.

ID	t(corr)[1]	t(corr)[2]	Total score
polyphyllin II	0.200	0.331	12.154
polyphyllin VII	0.319	0.282	10.433
Total steroid saponins	0.192	0.223	8.226
29#	−0.237	0.209	7.501
1#	0.442	−0.208	7.363
polyphyllin I	0.121	0.141	5.203
3#	−0.341	−0.137	5.148
31#	0.089	0.110	4.048
13#	0.481	0.104	4.039
20#	−0.744	−0.056	2.424
34#	0.541	0.032	1.429
23#	0.999	−0.032	0.667
42#	−0.879	−0.006	0.663
4#	−0.386	−0.008	0.470

Note: Total = ABS ((t(corr)[1]*50.3%)+(t(corr)[2]*36.4%)), the first principal component explained 50.3% of total information, and the second principal component explained 36.4% of total information.

From Table 3 and Table 4, we could calculate the average total score of different geographical origins (8.13) was much higher than different species (4.98) of *Paris*. The results suggested that different geographical origins had a much greater influence on *Paris* compared with different species. The complex geographical conditions, such as elevation, temperature, rainfall, sun exposure time, light quality and soil type are closely associated to different geographical origins environment.

## Conclusions

In conclusion, the results demonstrated that the combination of NIR spectrum and HPLC- based active components with multivariate analysis could be a powerful method for discriminating *Paris* of different origins and different species. The PLS-DA model showed different origins had a greater effect on *Paris* than different species. The quality of *Paris* showed regional dependence. A further study using NIR and HPLC-based metabolic profiling coupled with multivariate analysis would extend the coverage of the metabolites of *Paris* and provide the authoritative biomarkers responsible for the discrimination of *Paris* from different geographical areas. On the other hand, the metabolic profiling of different species of *Paris* also needs further study for providing basis of extending source of medicine-botanical origins.
